# A Standardized Framework for Fluorescence*-*Guided Margin Assessment for Head and Neck Cancer Using a Tumor Acidosis Sensitive Optical Imaging Agent

**DOI:** 10.1007/s11307-021-01614-z

**Published:** 2021-05-24

**Authors:** Pieter Jan Steinkamp, Floris Jan Voskuil, Bert van der Vegt, Jan Johannes Doff, Kees-Pieter Schepman, Sebastiaan Antonius Hendrik Johannes de Visscher, Wendy Kelder, Yalia Jayalakshmi, Jinming Gao, Baran Devrim Sumer, Gooitzen Michell van Dam, Max Johannes Hendrikus Witjes

**Affiliations:** 1 Department of Surgery, Nuclear Medicine and Molecular Imaging, University of Groningen, University Medical Center Groningen, PO Box 30.001, 9700 RB Groningen, The Netherlands; 2 Department of Oral & Maxillofacial Surgery, University of Groningen, University Medical Center Groningen, PO Box 30.001, 9700 RB Groningen, The Netherlands; 3 Department of Pathology & Medical Biology, University of Groningen, University Medical Center Groningen, PO Box 30.001, 9700 RB Groningen, The Netherlands; 4 Department of Surgery, Martini Ziekenhuis, Groningen, The Netherlands; 5 OncoNano Medicine Inc., Dallas, TX USA; 6 Department of Pharmacology, University of Texas Southwestern Medical Center, Dallas, TX USA; 7 Department of Otolaryngology Head and Neck Surgery, Simmons Comprehensive Cancer Center, University of Texas Southwestern Medical Center, Dallas, TX USA; 8 Tracer Europe B.V./AxelaRx B.V., Groningen, The Netherlands

**Keywords:** Fluorescence-guided surgery, pH activation, Tumor type-agnostic imaging, Margin assessment, Standardization

## Abstract

**Purpose:**

Intra-operative management of the surgical margin in patients diagnosed with head and neck squamous cell carcinoma (HNSCC) remains challenging as surgeons still have to rely on visual and tactile information. Fluorescence-guided surgery using tumor-specific imaging agents can assist in clinical decision-making. However, a standardized imaging methodology is lacking. In this study, we determined whether a standardized, specimen-driven, fluorescence imaging framework using ONM-100 could assist in clinical decision-making during surgery.

**Procedures:**

Thirteen patients with histologically proven HNSCC were included in this clinical study and received ONM-100 24 ± 8 h before surgery. Fluorescence images of the excised surgical specimen and of the surgical cavity were analyzed. A fluorescent lesion with a tumor-to-background ratio (TBR) > 1.5 was considered fluorescence-positive and correlated to standard of care (SOC) histopathology.

**Results:**

All six tumor-positive surgical margins were detected immediately after excision using fluorescence-guided intra-operative imaging. Postoperative analysis showed a median TBR (±IQR) of the fluorescent lesions on the resection margin of 3.36 ± 1.62. Three fluorescence-positive lesions in the surgical cavity were biopsied and showed occult carcinoma and severe dysplasia, and a false-positive fluorescence lesion.

**Conclusion:**

Our specimen-driven fluorescence framework using a novel, pH-activatable, fluorescent imaging agent could assist in reliable and real-time adequate clinical decision-making showing that a fluorescent lesion on the surgical specimen with a TBR of 1.5 is correlated to a tumor-positive resection margin. The binary mechanism of ONM-100 allows for a sharp tumor delineation in all patients, giving the surgeon a clinical tool for real-time margin assessment, with a high sensitivity.

**Supplementary Information:**

The online version contains supplementary material available at 10.1007/s11307-021-01614-z.

## Introduction

Optimal surgical management of head and neck squamous cell carcinoma (HNSCC) requires a tumor resection with adequate surgical margins as inadequate surgical margins are associated with an increased chance of local recurrence and metastasis, eventually causing reduced overall survival [1–3]. Fluorescence-guided surgery (FGS) is a novel imaging technique that allows tumor visualization during surgery by targeting tumor-specific biomarkers, proteins, or receptors. Multiple clinical phase 1 and phase 2 studies have investigated a variety of fluorescent imaging agents to enhance tumor detection, showing the potential of tumor-targeted FGS to guide surgical decision-making in a variety of clinical settings [4, 5]. However, the clinical translation of fluorescent-guided margin assessment lacks a standardized imaging method that allows immediate and reliable feedback for the surgeon. In this study, we provide an *ex vivo* specimen-driven imaging methodology in patients with HNSCC by implementing a promising pH dependent, tumor type-agnostic imaging agent called ONM-100 [6]. We suggest that *ex vivo* imaging might be preferable over *in vivo* imaging since external factors influencing imaging results are limited. Subsequently, we hypothesize the potential clinical effects of this standardized method for our patient cohort.

In recent years, a variety of tumor-specific fluorescence optical imaging agents have been developed. Different targeting strategies, like fluorophores conjugated to monoclonal antibodies (MoAb) or PARP1 inhibitors, have been clinically evaluated in HNSCC [5, 7–9]. Despite interesting results, target heterogeneity within cancer and target expression on the majority of healthy cells might cause false-positive signal and a limited discriminative strength [10]. Moreover, as the half-life of a MoAb is on average 7–10 days, administration needs to be done 2–5 days prior to surgery, which comes with logistical challenges [5, 11]. The optical imaging agent ONM-100, consisting of micelles containing indocyanine green (ICG), can overcome these limitations by targeting more ubiquitous tumor characteristics, namely tumor acidosis [12]. The mechanism of action of the imaging agent is described previously [12]. Briefly, the micelles dissociate in the acidic tumor microenvironment, allowing the ICG to un-quench and become fluorescent. As non-tumor tissue has a physiological pH, no “background” fluorescence activation is observed in non-tumor tissue, resulting in high tumor-to-background ratios (TBRs). The working mechanism has recently been clinically evaluated in 30 patients, showcasing the applicability of ONM-100 in four different tumor types [6].

As the field of FGS is rapidly expanding, data collection and implementation of fluorescence-guided surgery into the clinical workflow needs to be standardized, since results are highly affected by factors other than the distribution of the fluorophore itself. Therefore, we validate a novel framework using a pH-activatable optical imaging agent in HNSCC (Supplemental Figure S1). We hypothesize that our standardized framework with a specimen-driven approach, combined with the next-generation pH-activatable optical imaging agent, allows for reliable *real-time* intra-operative decision-making. In this study, we present data of 13 HNSCC patients who received ONM-100 24 h prior to surgery. Our data support the potential clinical value of this standardized framework using ONM-100 to alter *real-time* clinical decision-making, since all tumor-positive surgical margins were detected immediately after excision in the *ex vivo* environment.

## Materials and Methods

### Study Design

This study was conducted at the University Medical Center Groningen (UMCG) and data was analyzed retrospectively. The clinical study was approved by the Institutional Review Board (IRB number 2017/580) of the UMCG. Patients with histopathologically proven head and neck squamous cell carcinoma (HNSCC) scheduled for surgical removal were enrolled as part of a larger clinical trial (the Netherlands National Trial Register 7085, EudraCT number 2017-003543-38) assessing the safety and generic applicability and optimal dose of ONM-100 [6]. All patients gave their informed consent after being informed about the study. In the current study, we investigated if ONM-100 was suitable for intra-operative margin assessment in HNSCC surgery using a standardized fluorescence workflow (Supplemental Figure S2).

### ONM-100 Conjugation

ONM-100 consists of polymeric micelles labeled with indocyanine green (ICG), as described previously [12]. ONM-100 was administered intravenously 24h ± 8 h before surgery in different dosages, which has been extensively described in our previous study [6].

### Standardized Method for Fluorescence-Guided Clinical Decision-Making

#### Specimen-Driven Margin Assessment

The excised surgical specimen of all patients was retrospectively analyzed using the PEARL-trilogy® imaging device (Li-COR BioSciences Inc., Lincoln, NE, USA). This closed-field device is suitable for *ex vivo* tissue imaging, as it eliminates ambient light and enables standardization of imaging between specimens. The tumor and all resection planes of the specimen were imaged immediately (< 10 min) after tumor excision. The tissue thickness varied in size up to 10 cm. Imaging resolution was 85 μm. If a fluorescent lesion was detected at one of the resection planes, this lesion was manually delineated and considered fluorescence-positive if a TBR of > 1.5 was obtained. This was based on an earlier reported study which determined 1.5 as adequate for discrimination between tumor and non-tumor tissue in FGS [13]. The fluorescent lesion was macroscopically delineated and the remaining, non-fluorescent, area of the respective resection plane was considered as background to determine the mean fluorescence intensity (MFI). For each patient, MFI lesion/MFI background was used to determine the TBR on the resection planes. If a fluorescent lesion was detected, the location of the fluorescent lesion was correlated based on anatomical information with fluorescence images of the surgical cavity. For orientation of the surgical specimen, sutures were applied on the specimen to allow for maximal orientation, as per standard of care. For one patient, the specimen was too large for the sample stage of the PEARL-trilogy® imaging device. For this patient, the SurgVision Explorer Air® Vault was used (details described below).

#### Surgical Cavity Driven Margin Assessment

For the detection of remaining tumor in the surgical cavity, fluorescence imaging using two intra-operative camera devices, the SurgVision Explorer Air® (SurgVision B.V., Groningen, the Netherlands) and the NovaDaq Spy Elite (Stryker, Kalamazoo, MI, USA), was performed *in vivo*. All additional fluorescent lesions detected prior to surgery or after surgery in the surgical cavity were biopsied, based on the definition of TBR > 1.5, and analyzed for final histopathological examination if deemed safe and feasible by the attending surgeon. *In vivo* imaging took approximately 5–10 min per procedure.

#### *Ex vivo* Correlation of Intra-operative Fluorescence Results

To validate fluorescence imaging results, fluorescence results were correlated with final histopathology. Following SOC, the surgical specimen was formalin-fixed for at least 24 h and subsequently sliced in approximately 0.5 cm thick tissue slices. In the case a fluorescent lesion was detected on the surgical margin during imaging of the excised specimen, this was precisely correlated to the representative tissue slice. Next, tissue slices were embedded into formalin fixed paraffin embedded (FFPE) blocks according to SOC by a pathologist blinded for fluorescence results. Fluorescent areas at the tissue slices that were not initially selected for histopathological analysis by the blinded pathologist were additionally embedded. Histopathological assessment on hematoxylin and eosin (H&E) stained 3 μm tissue sections was done by a board-certified pathologist, blinded for fluorescence, and according to SOC.

### Fluorescence Imaging Devices

*In vivo* fluorescence images were obtained using two intra-operative cameras, namely the Explorer Air® (SurgVision B.V., Groningen, the Netherlands) or the Novadaq SPY Elite® (Stryker, Kalamazoo, MI, USA). Fluorescence imaging with the SurgVision Explorer Air® was performed as previously described by our group [5]. The Novadaq Spy Elite contains a light emitting diode (LED) with an excitation wavelength of 805 nm, designed for the detection of ICG. White light images and fluorescence videos were obtained during surgery. The working distance above the surgical field was 30 cm. Fluorescence gain could be manually adapted during imaging.

The excised whole specimen and tissue slices were imaged using the PEARL-trilogy® imaging device, using a CCD camera in the NIR wavelength (peak exCitation 785 nm, peak emission 820 nm). The field of view of 11.2 cm × 8.4 cm and the focus point can be adjusted based on specimen height. All images were stored in TIFF format and post-processed by applying a color scheme for the benefit of fluorescence signal intensity visualization and analyzed using Fiji (version 2.0.0-rc-68/1.52h). The fluorescence values did not change by applying the color scheme.

## Results

Thirteen patients with histopathologically proven HNSCC were included. Patient and fluorescence imaging characteristics are depicted in Table 1.

**Table 1.** Patient characteristics

Patient number	Age	Location of tumor	Tumor stage	Margin status	Distance to closest margin	Whole specimen fluorescence	Surgical cavity fluorescence
Patient 01	50	Mandible	pT4N1M0	Close	3.5 mm	-	-
Patient 02	69	Floor of mouth	pT3N2bM0	Positive	Cut through	+	-
Patient 03	53	Tongue	pT3N0Mx	Close	3 mm	+	+
Patient 04	46	Floor of mouth	pT3N0Mx	Positive	< 1 mm	+	-
Patient 05	80	Cheek	pT1N0Mx	Positive	Cut through	+	-
Patient 06	79	Cheek	pT2N0Mx	Close	3 mm	-	-
Patient 07	70	Tongue	pT3N2bMx	Positive	< 1 mm	+	+
Patient 08	48	Floor of mouth	pT1N1Mx	Free	> 5 mm	+	-
Patient 09	69	Mandible	pT2N0Mx	Positive	< 1 mm	+	+
Patient 10	85	Mandible	pT4N0Mx	Positive	Cut through	+	+
Patient 11	84	Palate	pT1N0Mx	Close	3 mm	+	-
Patient 12	58	Tongue	pT1N0Mx	Close	2 mm	+	-
Patient 13	77	Tongue	pT2N0Mx	Free	> 5 mm	-	-

Whole specimen fluorescence: presence of a fluorescent lesion at the edge of the resected specimen. Surgical cavity fluorescence: presence of a fluorescent lesion in the surgical cavity during surgical excision. + = positive for fluorescence, - = negative for fluorescence

### *Ex vivo* Specimen-Driven Margin Assessment

**Fig. 1.**
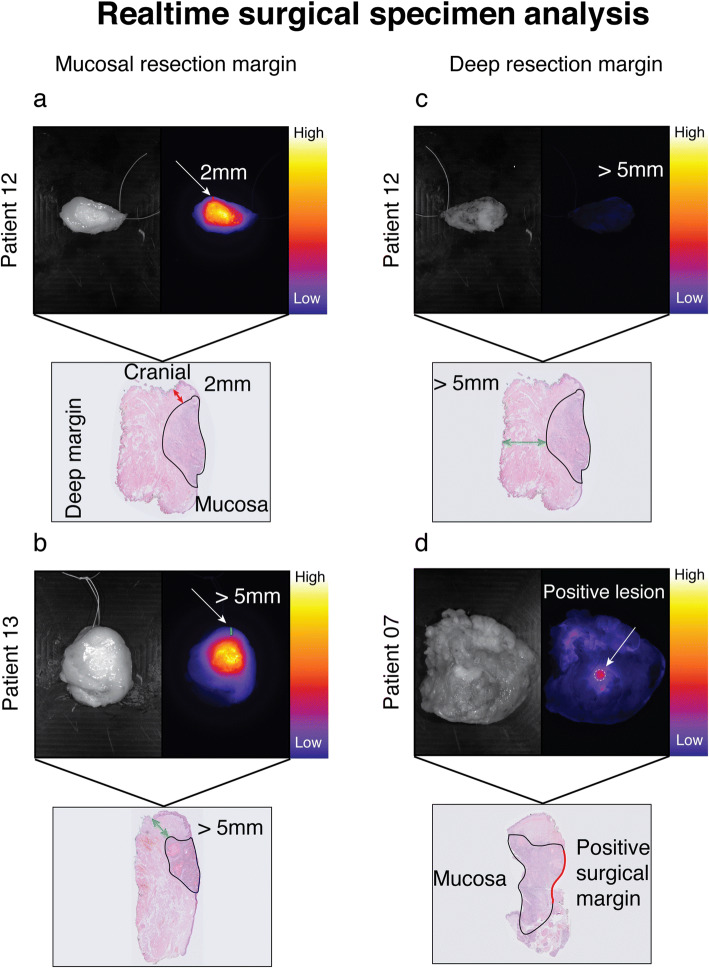
Real-time surgical specimen analysis. Fluorescence-guided analysis of the mucosal tumor and of the deep resection margins in HNSCC patients was performed using Li-COR PEARL Trilogy. **a** Representative image of a mucosal tongue tumor with an insufficient superficial surgical margin (2 mm). **b** Representative image of a mucosal tongue tumor with a sufficient superficial surgical margin (> 5 mm). **c** Representative image of a deep surgical resection margin negative for fluorescence which correlated with a tumor-negative margin. **d** Representative image of a positive fluorescent lesion on a deep resection margin correlating with a tumor-positive surgical margin.

**Fig. 2.**
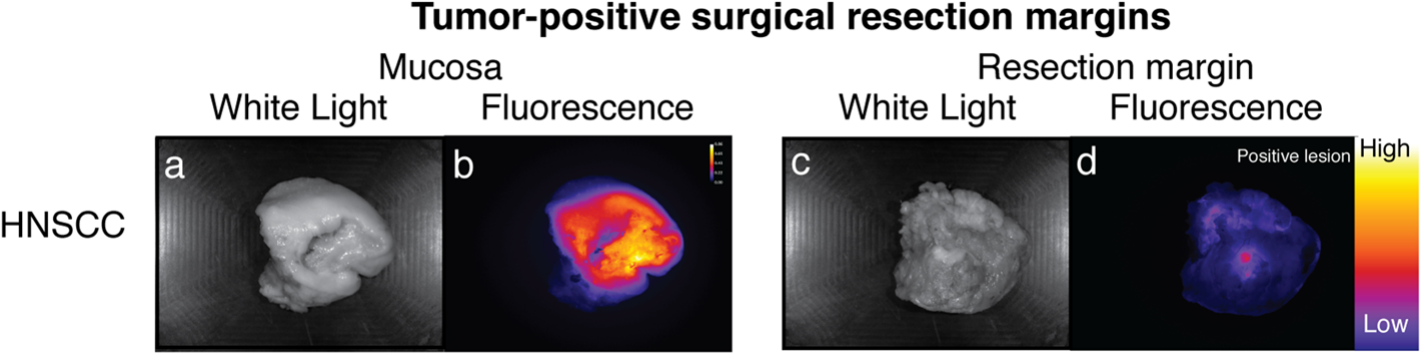
Tumor-positive surgical margins. Representative images of an excided tumor specimen, visualized on the mucosal site (**a**–**b**) which shows clear delineation of the tumor by fluorescence imaging. Next, the deep resection margin (i.e., basal surface) is shown (**c**–**d**), illustrating a sharply delineated fluorescence-positive lesion which correlated with a tumor-positive surgical margin of the respective specimen.

Both the mucosal and the deep resection planes were evaluated by fluorescence imaging of the excised specimen (*N* = 13, Figs. 1 and 2). All six histopathologically proven tumor-positive surgical margins (viable tumor cells ≤ 1 mm of margin) were detected using our standardized *ex vivo* fluorescence imaging framework, which enables accessible and fast identification of sharply delineated fluorescent signals (6/6, 100 % sensitivity). Five out of these six tumor-positive surgical margins were located at the deep resection margin (Supplemental Figure S2). All fluorescent lesions correlated to the location of the histopathologically tumor-positive surgical margin with a median TBR of 3.36 ± 1.62 (Supplemental Figure S3). Additionally, five histopathologically proven close margins (viable tumor cells 1–5 mm of margin) were diagnosed in this study, of which three were determined as fluorescence-positive. Two were scored as fluorescence-negative, which had both a surgical margin ≥ 2 mm (Table 1). Important to state, all surgical margins that were assessed as fluorescence-negative had a surgical margin > 2 mm. Of the two specimens that were histopathologically diagnosed with tumor-negative surgical margins (> 5 mm), one specimen was scored as fluorescence-positive at one of the resection margins, which was found to be caused by localized fluorescence around the salivary glands*.* This particular case showed no elevated fluorescence signal in the surgical cavity (Table 1). Fluorescence of the salivary glands was only observed in this single patient.

### Surgical Cavity Driven Margin Assessment

**Fig. 3.**
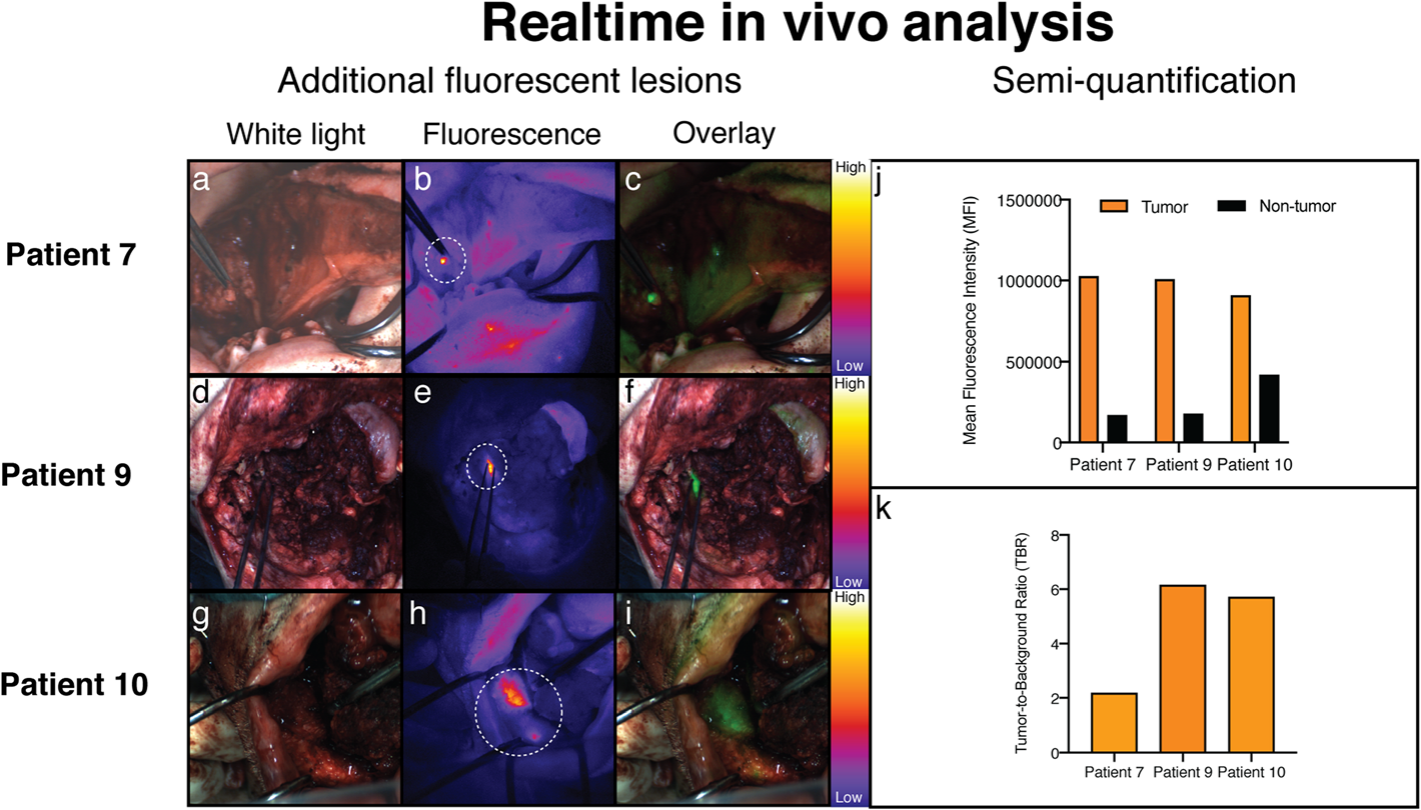
Real-time *in vivo* HNSCC analysis. Patient 7 with an in-transit metastasis detected after biopsy of a fluorescent spot in the surgical cavity (**a**–**c**). Patient 9 with a tumor in the mandible with a positive spot for fluorescence in the surgical cavity, which showed high-grade dysplasia without invasive carcinoma (**d**–**f**). Patient 10 with a tumor in the mandible with a clear fluorescent spot indicating a focal tumor-positive surgical margin as confirmed by histopathological assessment (**g**–**i**). Mean fluorescence intensity (MFI) of the fluorescent spot compared to the background for all three patients (**j**) and tumor-to-background ratios derived from the MFI data in all three patients (**k**).

During fluorescence imaging of the surgical cavity and peripheral surfaces in HNSCC (*N* = 13), four fluorescent lesions could be identified of which in three cases, a fluorescence-guided biopsy was performed. Three lesions were considered to be true positive. The mean TBR of these true positive biopsies was 4.68 (range 2.2–6.2) (Fig. 3). *Patient 7* underwent tumor excision of a HNSCC located at the tongue/floor of the mouth. After initial tumor excision, a fluorescent lesion was detected in the surgical cavity (TBR 2.2 Fig. 3, panels a–c). Histopathological analysis of the biopsy showed squamous cell carcinoma; however, there was no direct correlation with the location of the tumor-positive resection margin at the excised specimen. The tumor-positive biopsy is therefore diagnosed as in-transit metastasis. Despite adjuvant radiotherapy (cumulative dose 70 Gy) without chemotherapy due to severe comorbidity, the patient developed a recurrent tumor during radiotherapy located at the deep tongue musculature. During tumor resection of *patient 9*, fluorescence imaging of the medial side of the surgical area showed a fluorescence-positive lesion (TBR 6.2, Fig. 3 panels d–f). The biopsy of this lesion showed severe dysplasia without invasive carcinoma. Histopathological analysis of the surgical specimen revealed a tumor-positive surgical margin at the medial resection plane along with the presence of high-grade dysplasia, which correlated with the location of the fluorescent lesion in the surgical cavity. Lastly, the surgical cavity of *patient 10* showed a large area of fluorescence (TBR 5.7, panels g–i). Based on clinical arguments, related to the extent of the surgical defect, no biopsy was performed. The *ex vivo* fluorescence correlated with a focal tumor-positive surgical margin which corresponded to the location of the fluorescence in the surgical cavity (Supplemental Figure S2, panels q–t). In one case (patient 3), a false-positive fluorescence signal was observed in the surgical cavity (Supplemental Figure S4). Here, the lesion was suspected for perineural growth; however, the histopathology showed no tumor involvement nor any other reason for a specific ONM-100 activation.

### Real-Time Surgical Margin Determination — Defining the Room for Improvement

Three patients (patients 9, 11, 12) underwent an additional re-resection within a month after initial surgery according to SOC due to a tumor-positive or close surgical margin. During initial surgery of all three cases, the excised specimen was diagnosed as fluorescence-positive. Interestingly, during fluorescence imaging of the surgical cavity, no suspected lesions were detected in two of these cases (patients 11 and 12). This is congruent with the histopathological findings, showing a tumor-positive surgical margin (< 1 mm), but no cut through of the tumor, thus tumor cells within 0–1 mm of the border. As a result, histopathological analyses after the surgical re-resection in a second surgery showed no remaining tumor cells in these two HNSCC (re-)resected specimens. The remaining patients diagnosed with a tumor-positive or close resection margin positive for fluorescence (patients 2, 3, 4, 5, 7, 10) received no additional surgery based on clinical considerations such as severe comorbidity as decided within the multidisciplinary tumor board.

## Discussion

The field of fluorescence-guided surgery in oncology is expanding rapidly, but standardized methods to evaluate imaging results are not widely used. Our results indicate that a standardized *ex vivo* fluorescence-guided imaging method for margin assessment using the pH-activated imaging agent ONM-100 shows substantial clinical potential for *real-time* intra-operative decision-making. We identified all tumor-positive margins by detecting fluorescent lesions at the surgical resection margin with a tumor-to-background ratio > 1.5. This easy implementable framework, which can be implemented in the surgical theatre, can be used immediately after surgical excision allowing an alteration of the surgical strategy during initial surgery. Subsequently, we showed that ONM-100 can detect remaining tumor and severe dysplasia in the surgical cavity which was otherwise missed during standard of care surgery.

**Fig. 4.**
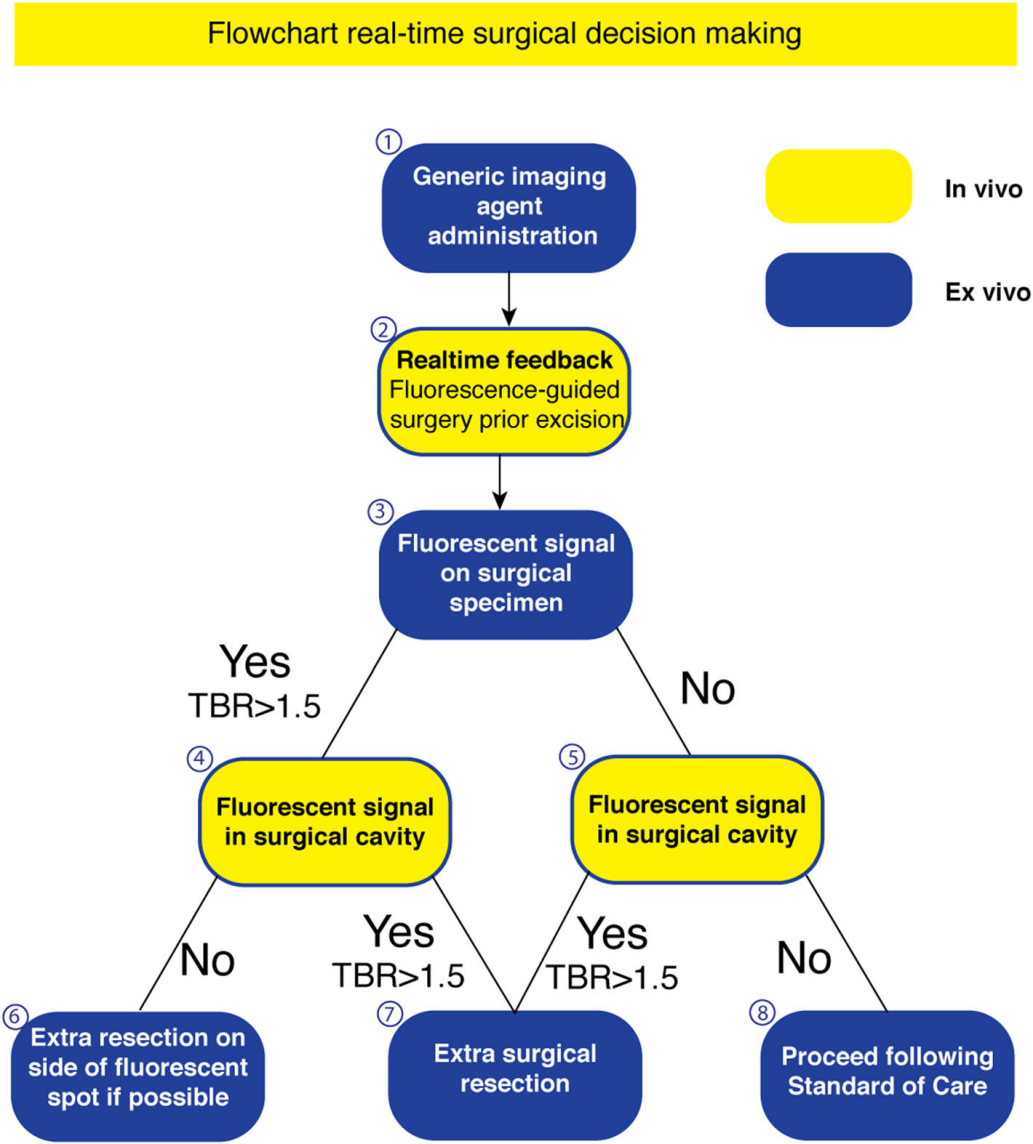
Flowchart real-time clinical decision-making. Suggested flowchart for fluorescence-guided surgery and surgical decision-making using fluorescence with this tumor generic fluorescence imaging agent. An additional surgical resection can be made in different scenarios when fluorescent spots with a tumor-to-background ratio of > 1.5 are observed on either the surgical specimen or in the surgical cavity.

We suggest the use of our standardized fluorescence framework, combined with ONM-100, for *real-time* surgical margin assessment in HNSCC and surgery (Fig. 4). Briefly, when a fluorescent lesion at one of the resection margins is detected (threshold TBR > 1.5), and if anatomical borders allow an additional resection *in situ*, an immediate re-resection can be considered [13]. This imaging procedure can be performed in the surgical theatre to improve surgical outcome. After tumor excision, imaging of the surgical cavity is performed subsequently to fluorescence imaging of the freshly excised surgical specimen which can guide the surgeon in performing an immediate re-resection. Additionally, fluorescence imaging of the surgical cavity can detect occult tumor and/or dysplastic lesions, whether or not related to the primary tumor, as highlighted by the observations in our cohort, in which one occult additional tumor lesion and a dysplastic lesion were found using fluorescence imaging of the surgical cavity. We show that FGS using ONM-100 could guide the surgeon to perform an immediate re-resection and potentially prevent (unnecessary) additional re-resections or adjuvant treatment. This potential is underlined by the fact that after an initial tumor-positive surgical margin, two HNSCC re-resections in this series were negative for residual tumor. In the unfortunate event that it is not possible to perform an additional resection, which accounts not only for head and neck cancer patients, the surgeon receives immediately feedback on the clinical situation and is able to alter the surgical plan (i.e., no further surgery, marking of positive spot for post-operative radiotherapy).

In the current study, intra-operative *ex vivo* fluorescence imaging for margin assessment has been prioritized above *in vivo* fluorescence imaging. *Ex vivo* specimen imaging using a closed-field and standardized imaging device allows for standardization of measurements, which is highly relevant in fluorescence-guided imaging where multiple factors like distance to the camera, angle of illumination, and environmental light can influence imaging results severely [14]. With the availability of a close-field imaging device at the surgical theatre, tissue can be imaged immediately after surgical excision allowing for direct surgical decision-making, as also has been described by other groups [11, 15, 16]. As the close-field imaging device used in the current study has a limited field of view, future developments in field of view, resolution, and imaging quality might further enhance intra-operative clinical decision-making.

Despite the investigated imaging agents showed promising results in previous studies, visual discrimination between tumor and non-tumor tissue remains challenging due to high, a specific background signals in non-tumor tissue [5, 7, 8, 12]. A low background signal, a consequence of the design of ONM-100, shows a clear and sharply delineated fluorescent signal in tumors in this study. This discriminative strength of ONM-100 is illustrated by the fact that the tumor-positive resection margins were clearly highlighted compared to the non-involved adjacent resection margin. Moreover, ONM-100 was administered within 24 h of surgery, providing major benefits over other fluorescence imaging agents which are administered 2–5 days prior to surgery [5, 7, 17]. Indeed, we have seen some false-positive fluorescence signal in salivary gland tissue, as is previously described as well [5]. The exact reason for this activation needs to be further analyzed in future studies; however, as we believe that a surgeon is able to differentiate salivary gland tissue from tumor tissue, we believe this might not be a significant clinical problem.

Based on our experience as a tertiary referral hospital, performing complex surgery often in late-stage HNSCC disease (Supplemental Figure S3), we identified three possible scenarios originating from the findings in this study following the detection of a fluorescent lesion. First, fluorescent lesions can be resected with an adequate margin without compromising vital structures. Second, an identified fluorescent lesion cannot be resected due to anatomical borders or the extent of disease. Third, new unidentified lesions not directly related to the initial tumor or irresectable lesions are identified which might change or cease the surgical plan. The first and last scenario occurred in this particular study. As this study shows, the fluorescent lesions detected at the excised specimen correlated in 100 % of the cases to tumor-positive resection margin and in 60 % of the cases to close resection margins. This implicates that, in theory, in ten patients, an immediate re-resection could be considered, thereby preventing a second surgery. This is highly relevant, since not all patients were eligible for second surgery due to their intrinsic comorbidities. From the possible ten re-resections, one re-resection would result in a case of overtreatment due to a false-positive fluorescent signal according to the current guidelines on tumor involved margins. If this eventually would lead to a higher local tumor control remains to be studied. We assume that in case of a successful immediate re-resection at the primary tumor site, adjuvant therapy can at least be de-intensified from the concomitant combination of chemoradiation to adjuvant radiotherapy alone. We therefore consider these data encouraging for further exploring the use of ONM-100 for the purpose of intra-operative margin assessment in a larger phase II trial.

Recent studies in HNSCC suggest a cutoff point of 2 mm between a tumor-positive and a tumor-negative surgical margin, as > 2 mm margin shows no significant increase in disease-specific survival [3, 18]. Therefore, a method that could discriminate between tumor presence within 2 mm and > 2 mm of the surgical border would be suited for *real-time* surgical guidance in HNSCC patients. Interestingly, all fluorescence-negative surgical margins found in this study had a surgical margin of at least > 2 mm, again underlining the clinical applicability of this technique. It should be noted that the numbers of tumor-positive margins detected in the current study are outside the average norm [19]. However, a tertiary referral hospital (UMCG) receives delicate and late-stage disease patients which a high a priori chance for tumor-positive surgical margins.

In conclusion, this study demonstrates the potential added value of specimen-driven fluorescence imaging of the surgical specimen in HNSCC patients after administration of the optical imaging agent ONM-100. We found a 100 % sensitivity for tumor-positive surgical margin detection using this technique and showed the potential for the intra-operative detection of occult disease. ONM-100 is easy to implement into standard clinical care due to the clear visual discrimination between tumor and non-tumor tissue, therefore having great potential in assisting in clinical decision-making during HNSCC surgery, preventing under- and overtreatment.

## Supplemental Information

Supplemental Figure S1 **| Real-time Clinical Decision-Making Framework** ONM-100 was administered intravenously approximately 24h (±8h) hours prior to surgery. (2) Fluorescence-guided imaging of the surgical specimen was performed *ex vivo* directly after excision of the tumor using a closed-field imaging device for positive margin detection (3) fluorescence-guided imaging *in vivo* of the surgical cavity was performed for detection of positive margins and/or occult disease (4) During all phases of standard pathology processing, fluorescent images of the whole specimen and fresh tissue slices were obtained (5) Correlation of the fluorescent signal on whole specimen and with standard histopathology was performed based on H/E slices by a board-certified pathologist blinded for fluorescence. (PNG 187 kb) Supplemental Figure S2 **| Tumor-positive Head- and Neck Cancer Surgical Margins** All five tumor-positive surgical margins imaged directly after tumor excision with the PEARL Trilogy are depicted. A region of interest (ROI) was drawn around the fluorescent spot and of the remaining tissue of the whole surgical specimen (denominated as background) and the Mean Fluorescent Intensity (MFI) was calculated. TBR was calculated as MFI fluorescent spot / MFI background. The ROI of the fluorescent spot was correlated to histopathological assessment. All tumor-to-background ratios of the five patients with a tumor-positive surgical margin. (u) (PNG 2091 kb) Supplemental Figure S3 **| Clinical imaging of late-stage HNSCC disease** Representative examples of pre-operative imaging of HNSCC tumors included in the current study. (PNG 1348 kb) Supplemental Figure S4 **| False positive intra-operative lesion** Fluorescence imaging of the surgical cavity of patient 3, illustrating the false positive lesion in the surgical cavity as described in the manuscript (PNG 313 kb)
