# Supplementary figures and images for: A Standardized Framework for Fluorescence-Guided Margin Assessment for Head and Neck Cancer Using a Tumor Acidosis Sensitive Optical Imaging Agent
Source: Mol Imaging Biol. 2021 May 24;23(6):809–17. doi: 10.1007/s11307-021-01614-z (PMC8578180; doi:10.1007/s11307-021-01614-z)

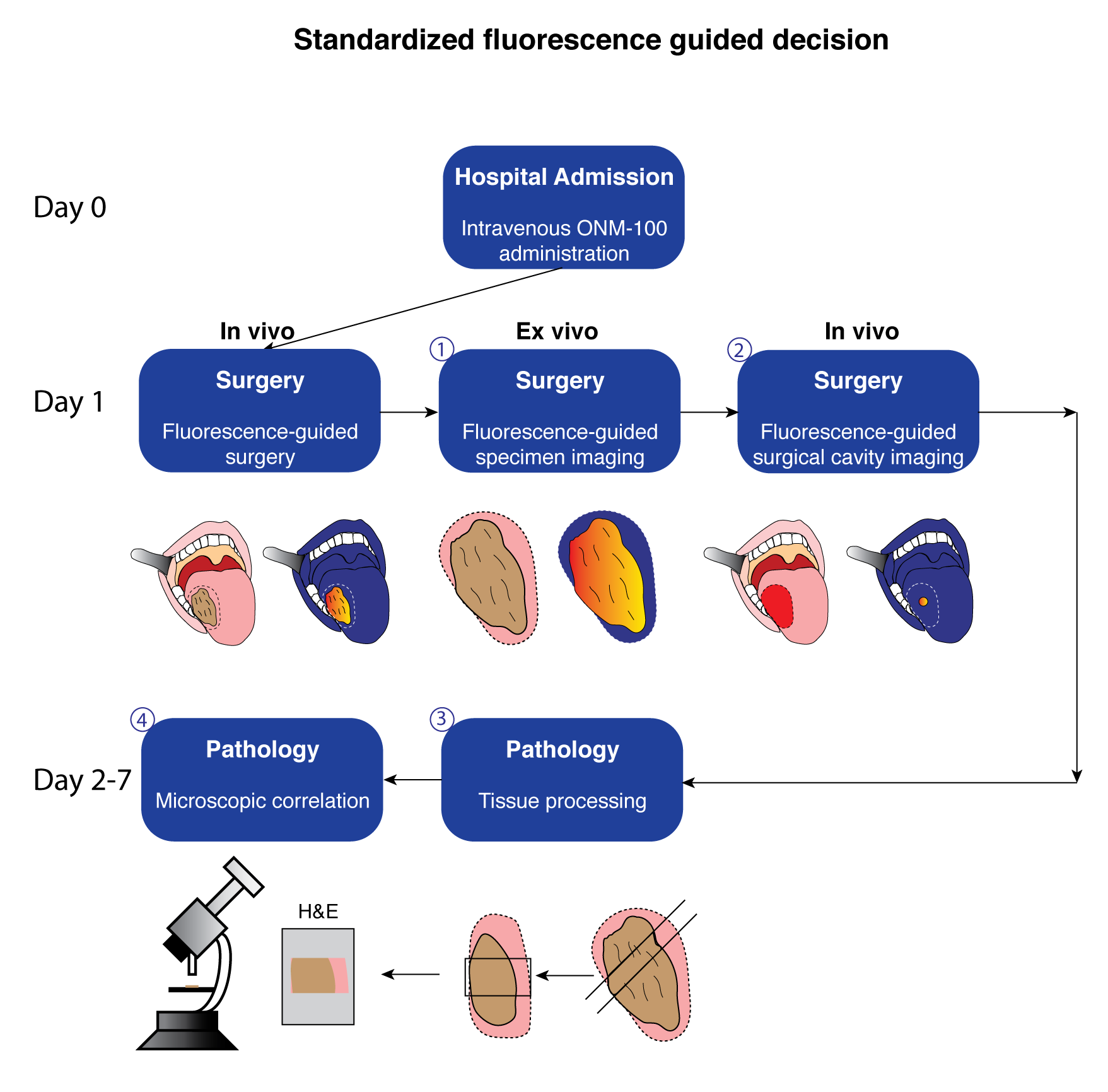

Supplement: Supplemental Figure S1 — | Real-time Clinical Decision-Making Framework ONM-100 was administered intravenously approximately 24h (±8h) hours prior to surgery. (2) Fluorescence-guided imaging of the surgical specimen was performed ex vivo directly after excision of the tumor using a closed-field imaging device for positive margin detection (3) fluorescence-guided imaging in vivo of the surgical cavity was performed for detection of positive margins and/or occult disease (4) During all phases of standard pathology processing, fluorescent images of the whole specimen and fresh tissue slices were obtained (5) Correlation of the fluorescent signal on whole specimen and with standard histopathology was performed based on H/E slices by a board-certified pathologist blinded for fluorescence. (PNG 187 kb) [file 11307_2021_1614_MOESM1_ESM.png]

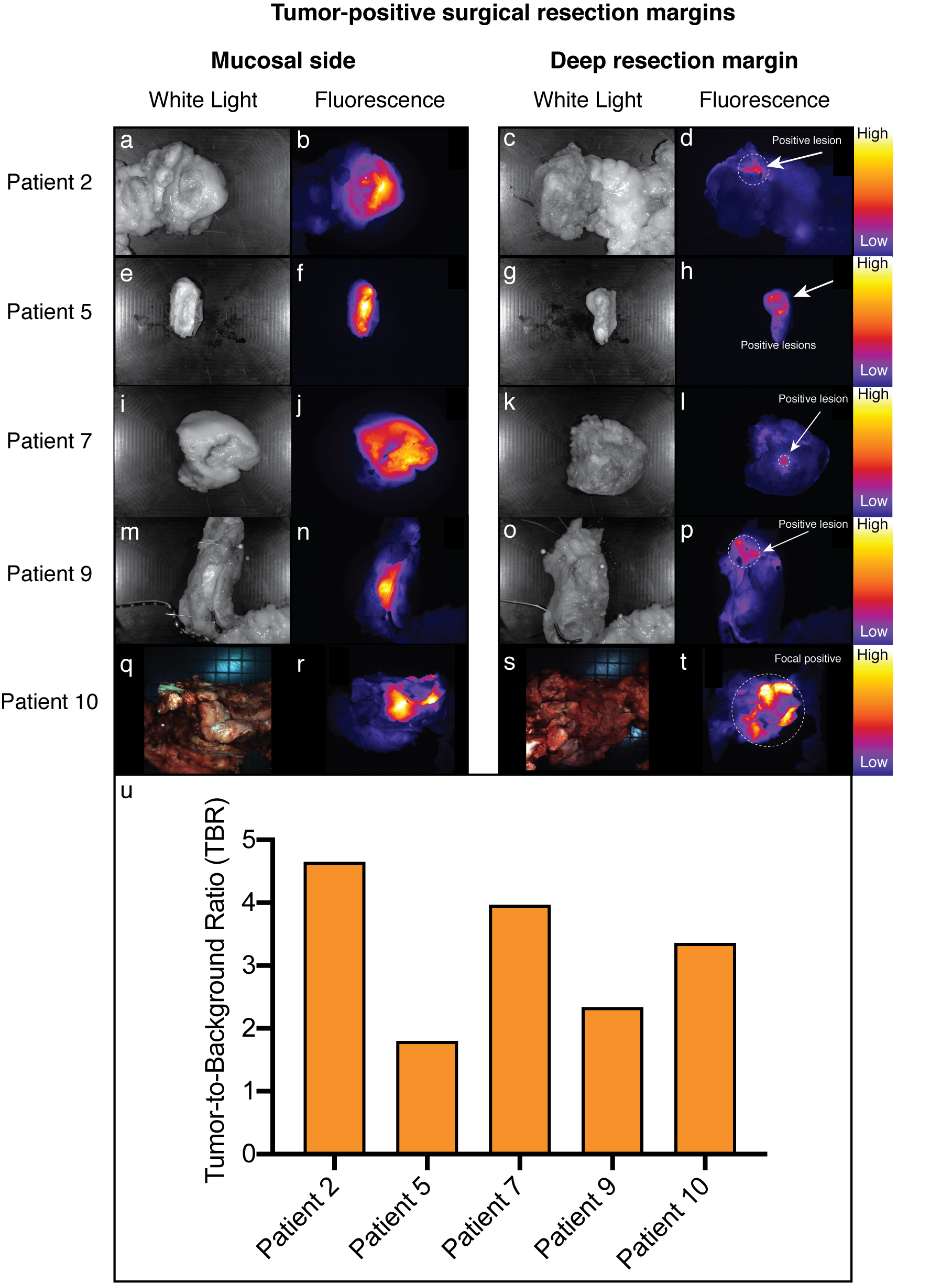

Supplement: Supplemental Figure S2 — | Tumor-positive Head- and Neck Cancer Surgical Margins All five tumor-positive surgical margins imaged directly after tumor excision with the PEARL Trilogy are depicted. A region of interest (ROI) was drawn around the fluorescent spot and of the remaining tissue of the whole surgical specimen (denominated as background) and the Mean Fluorescent Intensity (MFI) was calculated. TBR was calculated as MFI fluorescent spot / MFI background. The ROI of the fluorescent spot was correlated to histopathological assessment. All tumor-to-background ratios of the five patients with a tumor-positive surgical margin. (u) (PNG 2091 kb) [file 11307_2021_1614_MOESM2_ESM.png]

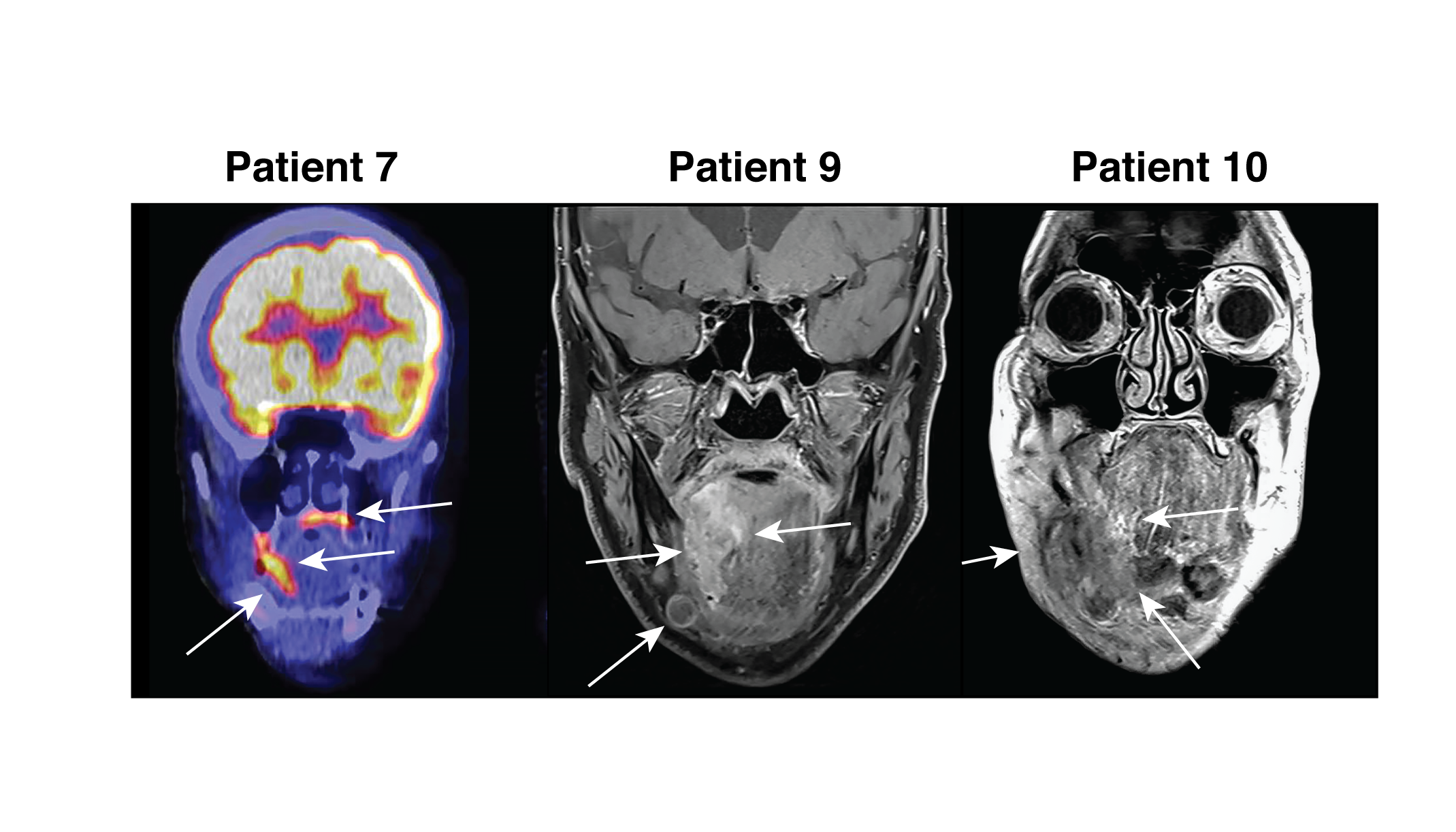

Supplement: Supplemental Figure S3 — | Clinical imaging of late-stage HNSCC disease Representative examples of pre-operative imaging of HNSCC tumors included in the current study. (PNG 1348 kb) [file 11307_2021_1614_MOESM3_ESM.png]

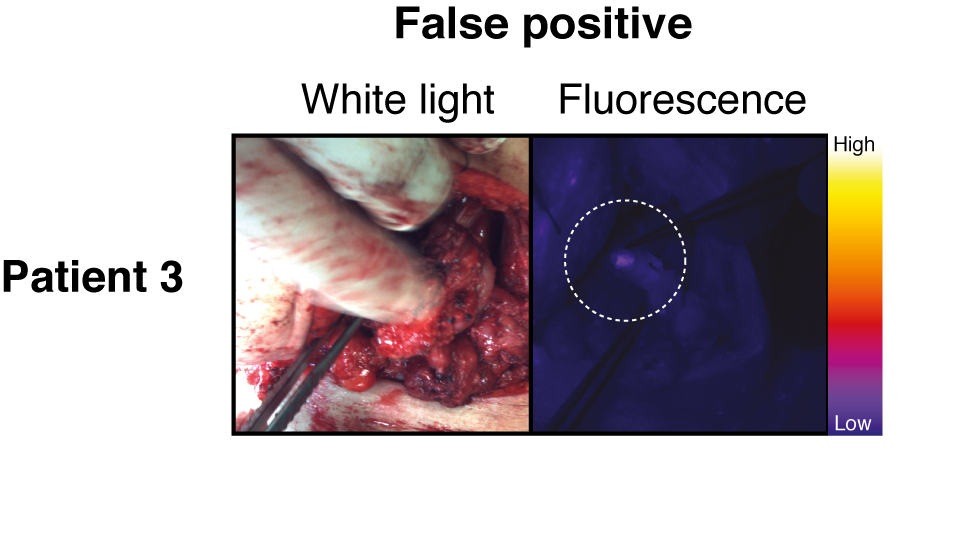

Supplement: Supplemental Figure S4 — | False positive intra-operative lesion Fluorescence imaging of the surgical cavity of patient 3, illustrating the false positive lesion in the surgical cavity as described in the manuscript (PNG 313 kb) [file 11307_2021_1614_MOESM4_ESM.png]
